# The Italian START-Register on Anticoagulation with Focus on Atrial Fibrillation

**DOI:** 10.1371/journal.pone.0124719

**Published:** 2015-05-22

**Authors:** Emilia Antonucci, Daniela Poli, Alberto Tosetto, Vittorio Pengo, Armando Tripodi, Nicola Magrini, Francesco Marongiu, Gualtiero Palareti, START-Register

**Affiliations:** 1 Department of Experimental and Clinical Medicine, Thrombosis Centre, University of Florence, Florence, Italy; 2 Department of Heart and Vessels, Thrombosis Centre, Azienda Ospedaliero-Universitaria Careggi, Florence, Italy; 3 Department of Hematology, Vicenza S. Bortolo Hospital, Vicenza, Italy; 4 Department of Clinical Cardiology, Thrombosis Centre, University of Padua, Padua, Italy; 5 Angelo Bianchi Bonomi Hemophilia and Thrombosis Center, Department of Clinical Sciences and Community Health, University of Milan, Milan, Italy; 6 Drug Evaluation Unit, WHO Collaborating Centre in Evidence-Based Research Synthesis and Guideline Development, Emilia Romagna Health and Social Care Agency, Bologna, Italy; 7 Department of Medical Sciences, University Hospital of Cagliari, Cagliari, Italy; 8 Coordinator of the START-Register, Bologna, Italy; Sapienza University of Rome, ITALY

## Abstract

START-Register – Survey on anTicoagulated pAtients RegisTer – is an independent, inception-cohort, observational, collaborative database aimed at recording prospectively the clinical history of adult patients starting anticoagulant treatment for any reason and using whatever drug. In this article we present the START-Register and give cross section baseline data focusing on non valvular atrial fibrillation (NVAF). Participants are asked to insert prospectively consecutive patients recorded as electronic file on the web-site of the registry. Required data are: demographic and clinical characteristics of patients, associated risk factors for stroke and bleeding, laboratory routine data, clinical indication for treatment, expected therapeutic range (in cases of treatment with vitamin K antagonists -VKAs). The follow-up is carried out to record: quality of treatment (for patients on VKAs), bleeding complications, thrombotic events, and the onset of any type of associated disease. To date 5252 patients have been enrolled; 97.6% were on VKAs because direct oral anticoagulants (DOAC) have been available in Italy only recently. The median age was 74 years [interquartile range (IQR) 64-80]; males 53.7%. This analysis is focused on the 3209 (61.1%) NVAF patients. Mean CHADS_2_ score was 2.1±1.1, CHADSVASc score was 3.1±1.3;median age was 76 years (IQR 70-81); 168 patients (5.3%) had severe renal failure [Creatinine clearance (CrCl) <30 ml/min]. Moderate renal failure (CrCl 30-59 ml/min) was found in 1265 patients (39.5%). The analysis of the START-Register data shows that two-third of patients who started chronic anticoagulant treatment had NVAF, one-third of them was > 80 years with high prevalence of renal failure.

## Introduction

Oral anticoagulation treatment with vitamin K antagonist (VKA) has been shown to be effective in numerous randomized clinical trials[1], but despite the potential clinical benefits they are underused especially in patients at high risk of bleeding [2]. In the last few years a good deal of effort has been directed towards the development of novel direct oral anticoagulants (DOAC) in order to avoid the difficulties and risks associated with VKA therapy. Several phase III, non-inferiority, large trials in patients with non-valvular atrial fibrillation (NVAF)[3–6], and studies of acute[7–11]or extended [12,13] treatment in patients with venous thromboembolism (VTE) have recently been published investigating the efficacy and safety of 4 different DOAC (dabigatran, rivaroxaban, apixaban and edoxaban) versus standard anticoagulation (in most cases VKAs) for preventing thromboembolic complications in these clinical conditions. In clinical trials however patients are usually carefully selected and there is very close control, factors that may probably result in lower rates of complications and therapeutic failure. This makes it difficult not only to assess the possible value and risk of DOAC in everyday clinical practice, but also to evaluate their health-economic benefits.

Observational studies offer information on clinical routine practice, and could highlight emerging issues. In 2011 we proposed the implementation of a collaborative database to prospectively record the clinical history of patients starting anticoagulant treatment for any reason and whatever the drug used. The study, called START-Register—Survey on anTicoagulated pAtients RegisTer—is designed to provide: a) information on the effects of available anticoagulant treatments, improving the knowledge of epidemiologic, diagnostic, and clinical features of thrombotic diseases; b) to enhance our understanding of the risk-benefits of the various anticoagulant drugs and therapy options. In this article we present the START-Register and its design, and give a cross section of baseline data collected at the moment, in particular focusing on included patients with NVAF.

## Material and Methods

### START-Register design

The START-Register is an observational, multicenter, dynamic cohort study that includes adults (≥18 years) who start anticoagulation therapy, whatever the drug and dosage used. More recently a branch of the registry also included patients treated with antithrombotic drugs, especially new antiplatelet drugs (START-Antiplatelet); the present article reports only on the anticoagulation branch of the registry. The aim of the START-Register is to collect data on the incidence of adverse events in patients taking anticoagulant, as well of determinants of them and quality of life or patient compliance to treatment. The study design and protocol has been drafted and approved by the Executive Committee that manages the START-Register. The Executive Committee designed a Coordinating Member (G.P.), who first obtained authorization to set up the registry from the Ethical Committee of his Institution (Azienda Ospedaliero-Universitaria, Policlinico S. Orsola-Malpighi, Bologna, Italy) on October 2011 (N = 142/2010/0/0ss") (NCT02219984). The same institution was entrusted to deploy and maintain the registry central database. The START-Register sets itself an indefinite time limit and is an entirely independent project. Public and private institutions, companies and individuals interested in the issue of anticoagulant treatment (manufacturers of drugs or other goods and services) are asked by the Executive Committee to help funding the registry via unrestricted grants without any right to access database data. Members of the Executive Committee do not receive any payment or fee for their work.

The registry is open to all physicians (called Participants) prescribing anticoagulant therapy and who agree to the Registry protocol. Participants should obtain approval from their local Institution Review Board. Participants are required to enter patients’ data only after obtaining a written informed consent from the patient. Participants can propose specific studies based on Registry data and have access to pertinent data after acceptance by the Executive Committee. Enrolment of at least 20 patients per year is required to be granted access to study data or propose collaborative studies.

### START-Register procedures

#### Patient eligibility

Patients ≥18 years who at the time of inclusion have been receiving anticoagulation therapy for no more than 30 days are eligible. The Registry aims at following patients especially during the first 12 months of treatment, though a long-term follow-up is recommended for patients who receive an indefinite anticoagulation. Therefore, patients with life-expectancy <6 months, or not residents in the Participant region, or planning leaving in the next 6 months are not eligible, as well as patients already enrolled in phase II or III clinical studies. Patients enrolled in other observational or phase IV studies can be included in the START-Register.

Follow-up of enrolled patients is mandatory for at least one year. Therefore, participants are required to provide data of all significant events occurring in enrolled patients for at least 12 months, including death, bleeding or thrombotic complications, and development of major organ failure even if use of anticoagulants has been interrupted before 12 months. Participants are required to enrol their patients consecutively, without any a-priori exclusion criteria other than life-expectancy or geographical inaccessibility. Definition of the time-framing for enrolment (e.g., one week every month, or the first month of the year) is left at each participant’s discretion, as long as it provides a random enrolment of patients. Between-participants difference in enrolled patients is anticipated, since some participants may be following patients having preferentially a disease (e.g., atrial fibrillation) or receiving only a particular anticoagulant drug. Participants-stratified analyses will be performed as required to account for these anticipated differences.

#### Baseline data

Patient’s clinical featuresare recorded by participants on web-based CRF (case report forms). Baseline data are demographic and clinical characteristics of patients, associated risk factors for stroke and bleeding, laboratory routine data, clinical indication for treatment, therapeutic range expected (in cases of treatment with VKA), use of concomitant drugs. Serum creatinine levels are measured by local hospital laboratories, and creatinine clearance (CrCl) is calculated by the Cockroft-Gault formula [14]. Renal failure was defined according to National Kidney Foundation stratification [15].

Patients with non-valvular AF are stratified for stroke risk evaluation according to CHADS_2_[16] and CHA_2_DS_2_VASc [17] scores while baseline bleeding risk is evaluated by using HAS-BLED score [18]. In patients with venous thromboembolism, the assessment of VTE site and presence of provoking factors is mandatory; presence of biochemical or molecular risk factors is optional.

#### Follow-up data

Participants are required to regularly follow-up enrolled patients at least quarterly, by phone call or ambulatory visit. An ambulatory follow-up visit is mandatory at least annually. Participants are required to provide detailed clinical reports of any relevant clinical outcome occurring in enrolled patients. For patients on VKA, time spent in the therapeutic range (TTR, computed according to the Rosendaal’s method [19]) is recorded every three months for the first year, annually thereafter.

#### Events

Relevant clinical outcomes are major bleeding complications (using the classification recommended by the International Society on Thrombosis and Haemostasis—[20]), any thrombotic event, development of cancer, organ failure, and death. A major bleeding event is defined as a bleeding event that is accompanied by a decrease in hemoglobin (Hgb) ≥ 2 g/dL, requiring transfusion with ≥ 2 units of packed red blood cells or occurring in a critical sites. Clinically relevant non-major bleeding event are classified as bleeding that does not satisfy the criteria for major bleeding but requiring hospital admission or a change in antithrombotic therapy. Minor bleeding events are all bleeding satisfactorily managed in an ambulatory setting. Diagnosis of stroke requires the abrupt onset of focal neurological symptoms lasting at least 24 hours and supported by congruent ischemic lesions at CT or MRI scan. Systemic embolism is defined by symptoms consistent with an acute loss of blood flow to a peripheral artery, which is supported by objective evidence of embolism. Acute MI is defined by thoracic symptoms and troponin elevation ≥ 2 x ULN or significant Q waves. Pulmonary embolism is diagnosed by detection of a new intraluminal filling on spiral CT scan, pulmonary angiogram or ventilation/perfusion lung scan (VPLS). Deep vein thrombosis is diagnosed by thrombus detection in a deep vein by abnormal compression ultrasound (CUS) or venography.

### Statistical Analysis

Descriptive analysis was performed. Continuous variables are expressed median and interquartile range (IQR) or as mean ±standard deviation(SD). Categorical variables are expressed as frequencies and percentages. The SPSS software for Windows, version 19 (SPSS Inc, Chicago, IL) was used for data processing.

## Results

The inclusion of patients in the registry started in January 2012; at the time of the present analysis (December 2013) 90 Participants, equally distributed in Northern, Central and Southern Italy, had enrolled 5252 patients. As shown in Table 1, males were slightly more represented (53.7%) than females; median age was 74 years and almost one-fourth of all patients were > 80 year old (24.2%). NVAF was the most frequent indication for treatment present in almost two-thirds of all patients (61.1%), followed by VTE (28.6%). VKAs were almost the only type of anticoagulant drug used (Table 1).

**Table 1:** Patients enrolled in the START-Register, indication for anticoagulation and type of treatment.

N.	5252
Males n (%)	2818(53.7)
Median age, y (interquartile range)	74 (64–80)
Patients aged > 75 years (%)	2302 (43.9)
Patients aged> 80 years	1268 (24.2)
*Indication for Anticoagulant treatment n (%)*	
Non-valvular atrial fibrillation	3209 (61.1)
Venous thrombembolism	1500 (28.6)
Heart prosthetic valves	223 (4.2)
Valvular atrial fibrillation	104 (2.0)
Other	216 (4.1)
*Type of treatment (%)*	
VKA	5130 (97.6)
DOAC	109 (2.1)
LMWH	13 (0.3)

VKA = vitamin k antagonist; DOAC = direct oral anticoagulant;

LMWH = light molecular weight heparin

The present analysis is focused on the baseline characteristics of the 3209 NVAF patients included in the registry, listed in Table 2.

**Table 2:** Clinical characteristics of NVAF patients included in the START-Register.

N.	3209
Median age, y (interquartile range)	76 (70,81)
Patients > 75 y (%)	1691 (52.7)
Patients 60–69 y	546 (17.0)
Patients 70–79 y	1373 (42.8)
Patients 80–89 y	1002 (31.2)
Patients ≥ 90 y	55 (1.7)
Males (%)	1770 (55.2)
Chronic NVAF (%)	1691 (52.7)
*Type of treatment*	
Warfarin	3070 (95.7)
Acenocoumarol	38 (1.2)
Dabigatran- Rivaroxaban	101 (3.1)
*Past medical history (%)*	
Heart failure	463 (15.0)
Hypertension	2673 (84.9)
Diabetes	639 (20.6)
Coronary Artery Disease	616 (19.9)
Peripheral Artery disease	169 (5.5)
Previous stroke/TIA	448 (14.6)
Previous major bleeding	64 (2.0)
*Other characteristics at inclusion (%)*	
CHADS_2_ score (mean±SD)	2.1±1.1
CHA_2_DS_2_VASc score (mean±SD)	3.0 ± 1.3
HAS-BLED score (mean±SD)	2.5± 1.0
Active cancer	76 (2.5)
Creatinine clearance <30 ml/min	168 (5.3)
Antiplatelet drugs + VKAs	532 (16.6)
Dual antiplatelet drugs + VKAs	50 (1.6)

VKA = vitamin k antagonist; TIA = Transient ischemic attack

More than half of the patients were > 75 year old, and about one third was 80 years or more. The distribution of patients stratified for stroke risk by using CHADS_2_ and CHA_2_DS_2_VASc score, and for bleeding risk by using HAS-BLED score is shown in Table 3.

**Table 3:** Clinical characteristics of AF patients in relation to stroke and bleeding risk stratification models.

Score	CHADS_2_ n(%)	CHA_2_DS_2_VASc n(%)	HAS-BLED* n (%)
0	196 (6.6)	59 (2.0)	77 (2.5)
1	842 (28.2)	267 (9.0)	362 (11.9)
2	1108 (37.1)	729 (24.6)	1024 (33.6)
3	496 (16.6)	981 (33.1)	1132 (37.1)
4	274(9.2)	532 (17.9)	409 (13.4)
5	63 (2.1)	301 (10.1)	45 (1.5)
6	5 (0.2)	84 (2.8)	1 (0.0)
7		14 (0.5)	
8		1 (0.0)	
9		/	

*all patient were naïve, no time in therapeutic range

Among the small group of 109 patients treated with DOAC, the median age was 76 years [interquartile range (IQR) 70–81]; the mean CHADS_2_ and CHA_2_DS_2_VASc scores were 2.2 ± 1.3 and 3.1 ± 1.3, respectively; and the mean HAS-BLED score was 2.5± 0.9. The association between VKA and an antiplatelet drug was present in 532 patients (16.6%), while other 50 anticoagulated patients were on double antiplatelet therapy (1.6%) (Table 2). The analysis of renal function showed that 168 patients (5.3%) had severe renal failure (CrCl<30 ml/min). Moderate renal failure (CrCl 30–59 ml/min) was found in 1265 patients (39.5%), and 329 patients in this group (10.3% of the whole cohort) had CrCl between 30 and 40 ml/min. Table 4 shows some clinical characteristics of NVAF patients enrolled in the study compared to those of patients enrolled in the phase III trials with DOAC and those included in the observational GARFIELD Registry [21].

**Table 4:** Some characteristics of NVAF patients enrolled in START-Register, compared to those in the GARFIELD Registry and in randomized trials on DOAC.

	START	Garfield [19]	RE-LY [3]	Rocket-AF [4]	Aristotle [5]	Engage AF [6]
**Age yrs**	74.6±9.6	70±11	72±9	73 (65,78)	70 (63,76)	72 (64,78)
**CrCl ml/min %**						
30–59	39.7	11.4				
30–50	23.9		19.3	20.8	15.1	19.3
< 30	5.3	2.0	Excluded	Excluded	1.5*	Excluded
< 15	0.02					
**BMI**	28.3±5.1	28±5	82.6 Kg ±19.9	28 (25,32)	82 Kg (70–95)	NA
**History of ACS %**	20	10	17	17	15	NA
**Diabetes %**	21	22	23	40	19.4	36.1
**CHADS**_**2**_ **score**	2.1±1.1	1.9±1.2	2.1±1.1	3.5±0.9	2.1±1.1	2.8±1.0

DOAC = direct oral anticoagulant

CrCl = creatinine clearance

Data are expressed as mean ± SD, or median (IQR), or %; NA = not available

* = patients with CrCl<25 ml/min were excluded

Finally, we estimated the number of NVAF patients who would have been compliant with the rules recently issued by the Italian Regulatory Drug Agency (AIFA) to receive from the National Health System complete reimbursement of treatment with one of the marketed DOAC. For VKA-naïve patients (as were the patients included in the registry), there were two criteria established by the Agency: a) the concurrent presence of two clinical characteristics: a CHA_2_DS_2_VASc score above a specific value that was different for the various drugs (see Table 5) and a HAS-BLED score > 3 (for all the drugs); or b) the impossibility of performing the VKA-therapy due to the presence of objective difficulties to comply with the need for the periodic laboratory controls (a condition that was not relevant in our series of patients). After exclusion of patients with severe renal insufficiency (following the criterion established by drug producers) the number of patients who would have been compliant with the two a) criteria was calculated. In line with the criterion of a HAS-BLED score > 3, which was the most restrictive, only about 15% of patients qualified for being treated with DOAC.

**Table 5:** Number of anticoagulation-naive patients with NVAF who at the moment of inclusion in the START-Register would have been compliant with the lower limit of creatinine clearance declared by the drug companies and with the criteria established from the Italian Regulatory Agency (AIFA), in order to be potentially treated with DOA. The criterion regarding the individual difficulties in performing the laboratory control of INR, as well as the percentage of time in range (all the START-Register patients were VKA-naive at inclusion) have not been taken into account for this evaluation.

	Dabigatran*	Rivaroxaban**	Apixaban¶
	patients eligible 3041	patients eligible 3195	patients eligible 3195
**CHA**_**2**_ **DS**_**2**_ **-VASc**	≥ 1 = 2746/3041 (90.3)	> 3 = 932/3195 (29.2)	> 2 = 1913/3195 (59.9%)
**HAS-BLED**	> 3 = 455/3041 (15.0)	> 3 = 455/3195 (14.2%)	> 3 = 455/3195 (14.2%)

CrCl = creatinine clearance

*after exclusion of 168 patients with CrCl< 30 ml/min

**after exclusion of 14 patients with CrCl< 15 ml/min

^¶^after exclusion of 14 patients with CrCl< 15 ml/min

## Discussion

This is the first paper presenting the Start-Register, an ongoing, collaborative, prospective, observational Register aimed at recording the clinical history of patients starting anticoagulant treatment, whatever the drug and dosage used and whatever the indication to treatment. Data reported in this paper describe the baseline characteristics of patients included after the first two years of activity of the Register, in particular focusing on those with NVAF who in the large majority received warfarin. In fact, only a small group of included patients were treated with DOAC. The DOAC currently available and reimbursable by the National Health System for treatment of NVAF (dabigatran, rivaroxaban, apixaban) have been marketed in Italy only during the last few months; furthermore the Italian Regulatory Drug Agency (AIFA) has established strict regulations to allow the prescription of a DOAC limiting in this way their use. Since the Participants in the Registry are equally distributed across Italy, the present inception-cohort can be considered representative of patients starting anticoagulation throughout Italy. Patients were relatively old, with just under half of them being > 75 years old at inclusion. Non valvular atrial fibrillation was the most frequent indication for anticoagulation (61.1%), followed by VTE (28.6%).

The analysis of NVAF subjects shows that the mean age of patients enrolled was slightly higher than that observed in the trials on DOAC and in the GARFIELD Registry [21]. One third of our patients were > 80 years old at inclusion; this confirms the advanced age of patients treated for this indication, this subpopulation is not sufficiently represented in phase III trials on DOAC (7–9,11). Other clinical characteristics of our cohort are comparable to those observed in patients enrolled in observational registries [21–23] Instead, our NVAF cohort shows a particularly high prevalence of moderate renal failure (CrCl 30–59 ml/min). As a matter of fact, moderate renal failure is present in about 40% of patients enrolled in the Registry compared with only 11% in GARFIELD’s cohort and 15–20% in trials with DOAC. In addition, the percentage of patients with severe renal failure, a group of patients excluded from the above trials, is higher than in the GARFIELD Registry, reaching 5% of our NVAF population. The presence of renal failure appears to be particularly important in the START Registry NVAF population. This finding can be in part explained by the older age of our population, that is represented for 45% by patients with ≥75 years. The cohort of patients with moderate renal failure is of particular clinical interest because patients are exposed to a rapid worsening of the renal function in case of intercurrent diseases, such as infections or heart failure. If treated with a DOAC this group of patients should be carefully monitored. DOAC are in fact mainly cleared via the kidneys—though with important differences between the available drugs—and tend to accumulate in case of kidney failure, with a subsequent increase in bleeding risk. In our cohort moderate renal failure is found in about 40% of patients, and about 10% have CrCl< 40 ml/min. It is worth noting that about 10% of patients treated with DOAC in our cohort are in this high risk group.

We are aware that our study has limitation. The participants to the Registry are mainly Anticoagulation Clinics and cardiologists, instead neurologists and general practitioners are less represented. However, consecutive patients enrolment is mandatory so limiting the selection of patients. In addition, participants in the Registry are geographically widely distributed across Italy, involving Centres of some small and main Italian cities.

Finally, it should be noted that, in accordance with the regulations established by the Italian Regulatory Drug Agency (AIFA), only a small portion (approximately 15%) of NVAF patients included in the Registry could have received the treatment with one of the available DOAC and reimbursed by the Italian National Health System. In fact, only a small portion of VKA-naïve NVAF patients proved to be compliant with the AIFA’s criteria for treatment reimbursement in. These criteria have been defined on the basis of the characteristics of patients enrolled in DOACs phase 3 trials, and not on the basis of cost-effectiveness analysis. However, this choice is questionable for several aspects. One of these is the indication to use DOAC in patients with a HAS-BLED score ≥3. These drugs have shown a safety performance at least similar to that of VKA, and in some cases even better, without any specific relationship with the HAS-BLED. Furthermore, two items that concur in the scoring, or are not available in VKA-naïve subjects (labile-INR, 1 point), or are conditions that may put at risk the use of DOAC (renal or liver function impairment, 1 point each). As regards the CHA_2_DS_2_VASc score evaluation, it is singular that the minimal level accepted for reimbursement differs so widely across the three available drugs. Unless the Agency is aware of results not available in the published trials, it is difficult to understand why patients should have a score of 4 or more to receive reimbursement with rivaroxaban, of 3 or more with apixaban, while a score of 1 is sufficient to be treated with dabigatran. For the above reasons, and for the need of fair and objective comparison of performances of different DOAC in a context of real life, we hope that these regulations can soon be changed by our Agency.

In conclusion, the analysis of baseline characteristics of the inception-cohort anticoagulated patients included in the START Register shows that almost two-third of patients started chronic anticoagulant treatment because of NVAF, and one-third of them is > 80 years at inclusion. The prevalence of renal failure is especially high in our NVAF population, since about 40% of patients have moderate and > 5% severe renal failure, the latter being an exclusion criterion in almost all the trials on DOAC. The START Register is now prospectively including non-selected new anticoagulated patients and collecting all the clinical data regarding their initial conditions and follow-up. The clinical use of DOAC started very recently in our country and the Registry will allow the clinical history of patients treated with conventional or newly proposed anticoagulant agents to be compared.
